# Word learning in the field: Adapting a laboratory-based task for testing in remote Papua New Guinea

**DOI:** 10.1371/journal.pone.0257393

**Published:** 2021-09-16

**Authors:** Karen E. Mulak, Hannah S. Sarvasy, Alba Tuninetti, Paola Escudero

**Affiliations:** 1 Australian Research Council Centre of Excellence for the Dynamics of Language, Canberra, Australian Capital Territory, Australia; 2 The MARCS Institute for Brain, Behaviour and Development, Western Sydney University, Milperra, New South Wales, Australia; 3 Department of Hearing and Speech Sciences, University of Maryland, College Park, Maryland, United States of America; 4 Department of Psychology, Bilkent University, Ankara, Turkey; Leiden University, NETHERLANDS

## Abstract

Adapting laboratory psycholinguistic methods to fieldwork contexts can be fraught with difficulties. However, successful implementation of such methods in the field enhances our ability to learn the true extent and limitations of human behavior. This paper reports two attempts to run word learning experiments with the small community of Nungon speakers in Towet village in the Saruwaged Mountains, Papua New Guinea. A first attempt involved running a cross-situational task in which word-object pairings were presented ambiguously in each trial, and an explicit word learning task in which pairings were presented explicitly, or unambiguously, in each trial. While this quickly garnered a respectable 34 participants over the course of a week, it yielded null results, with many participants appearing to show simple patterned responses at test. We interpreted the null result as possibly reflecting the unfamiliarity of both the task and the laptop-based presentation mode. In Experiment 2, we made several adjustments to the explicit word learning task in an attempt to provide clearer instructions, reduce cognitive load, and frame the study within a real-world context. During a second 11-day stay in the village, 34 participants completed this modified task and demonstrated clear evidence of word learning. With this success serving as a future guide for researchers, our experiences show that it may require multiple attempts, even by experienced fieldworkers familiar with the target community, to successfully adapt experiments to a field setting.

## Introduction

Psychological research disproportionally studies participants living in Western, educated, industrialized, rich, and democratic countries (so-called WEIRD societies) [1]. An analysis of studies published between 2003 and 2007 across six major journals of the American Psychological Association found that 96% of participants were from WEIRD societies, representing only 12% of the global population [2]. A follow-up investigation of studies published in the same journals between 2014 and 2018 found that this overall number remained unchanged [3]. Thus, despite a decade of awareness and discussion of this bias, little progress has been made improving this representational inequity.

**Fig 1:**
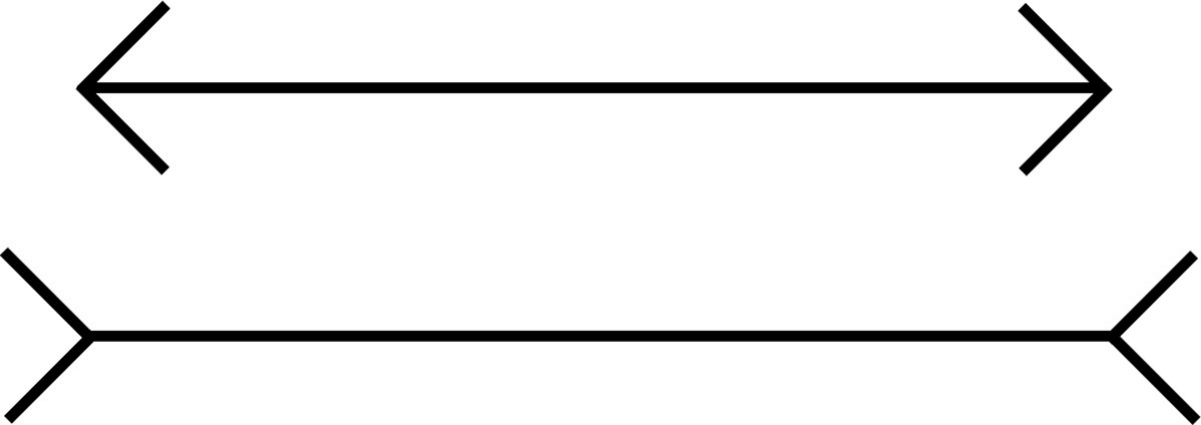
Depiction of the Müller-Lyer illusion [4]. Although the two horizonal lines are the same length, the bottom line appears longer to those who perceive the illusion.

This bias likely has had profound impact on the field, due to the propensity of researchers (who tend to come from WEIRD societies themselves [2,3]) to generalize or frame findings as reflecting universal aspects of human behavior and cognition. To illustrate this point, Henrich and colleagues [1] compiled examples across the behavioral sciences demonstrating differences between populations from industrialized vs. small-scale societies, between Western and non-Western societies, and even within Western, industrialized populations. For instance, many optical illusions are thought to arise from low-level visual processing, which would not be expected to vary across human populations. But they report work demonstrating that while Americans were susceptible to the Müller-Lyer illusion [4] (Fig 1), members of the non-industrialized, non-western San foragers of the Kalahari Desert were not [5]. It was through testing a wider range of community types that they could conclude that even what was thought to be a feature of basic visual perception can be influenced by cultural experience.

As a subfield of the behavioral sciences, psycholinguistics is not immune to this issue. The majority of psycholinguistic research has been conducted on speakers of only a handful of the world’s thousands of languages. This has led to some instances where replicated findings were speculated to represent universals of language processing, until testing speakers of underrepresented languages challenged that view. For instance, starting at around the time of their first birthday, infants brought up in French [6–10], Italian [11], and English [12–14] (though see [15]) language environments demonstrate a “consonant bias,” relying more on a word’s consonants than its vowels in lexical processing and word learning. Development of the consonant bias was thought to be an innate aspect of language development, but that idea was challenged when Danish-learning 20-month-olds demonstrated a vowel bias instead [16]. Unlike the languages that had been previously tested, Danish has more vowels than consonants [17], seemingly leading Danish infants to weight them as more informative in lexical processing. Thus, the development of the consonant bias, rather than being innate, appears to be shaped by the language environment of the listener [16].

In another example, the Possible Word Constraint (PWC) [18] is a proposed universal constraint on what can be a possible word, namely that a single consonant or cluster of consonants alone cannot. In continuous speech perception, words are segmented from the speech stream as they are recognized (see [19]), thus the PWC reduces the number of possible words during lexical selection, in turn making continuous speech processing more efficient. For instance, when hearing “wind,” “win” is not considered as a possible segmentation of the speech signal, since the remaining “d” violates the PWC. In “windy” or “window,” “win” can be a candidate in the lexical selection process since “dy” and “dow” do not violate the PWC. Experimental testing across a variety of languages (Cantonese [20], Dutch [21], English [18], French [22], German [23], Japanese [24], Sesotho [25], and Slovak [26]) have supported the PWC as a language universal. However, when tested in speakers of Tashelhit Berber, a North African language that—unlike the other languages tested—allows clusters of consonants to be words, such as [xdm] (meaning *work*), participants’ word segmentation did not appear to be governed by the PWC, challenging the notion that it is an immutable property of continuous speech perception [27]. Thus, restricting research to limited groups of people and languages has led us astray, not only in terms of incorrect conclusions about the generality or specificity of certain aspects of language learning and processing, but also by stopping us from considering a more complete range of the possibilities in human language processing.

Therefore, there is a clear need for and value in studying psycholinguistics across a broader range of languages and peoples. However, the practices of psycholinguistic research have been, in a sense, optimized for WEIRD settings. Laboratories have tended to study the languages and populations (often young university students) available in their community for reasons of convenience, familiarity, and relevance to their communities. The procedures and methods for data acquisition in psycholinguistics often involve written instruction or responses, and computer-based activities. They may be modeled on the multiple-choice-type tests that students in WEIRD educational systems begin taking at young ages. Data collection sessions are often lengthy, repetitive experiences in front of a computer screen, completing seemingly arbitrary or purposeless tasks, allowing the collection of large volumes of fine-grained data. In many non-WEIRD settings, these tasks are simply inappropriate, due to differences in literacy, computer use, and familiarity with a test-taking style of task. Beyond this, in some societies, assumptions about questions and answers may be very different from the neutral, objective role assumed in many experimental tasks [28]. For instance, it may be inappropriate to ask for opinions on something that the respondent did not personally experience [29].

Expanding research to a broader range of cultures and societies is clearly necessary, and requires bringing the lab to the field. This can be problematic when working with remote communities, who may be far less familiar with digital technology compared to the average Western university student. The remote field psycholinguist is thus tasked at the very least with adapting methods to the abilities of the remote community members, and ideally does this in a way that still allows for comparison with other populations. Here we document two attempts to adapt a psycholinguistic word learning experiment that can be administered in remote communities using minimal adaptations to the test phase. The goal was to create a word learning task appropriate for testing both in communities with members with limited literacy and familiarity with computer-based technologies, and in conventional laboratory settings, allowing for comparisons between populations.

For this endeavor, we recruited a community of speakers of the Nungon language, resident in Towet village of the Saruwaged Mountains in Papua New Guinea. In Experiment 1, which marked the community’s first time participating in a psycholinguistic experiment, we compared participants’ ability to learn novel associations between auditory words and two-dimensional line drawings in two types of commonly used word learning paradigms to determine whether one task is better suited for testing in a non-WEIRD population. The first was an explicit word learning paradigm (e.g., [30]) in which each novel object is shown explicitly, that is, individually in tandem with its corresponding word. The second was a cross-situational word learning (XSWL) paradigm (e.g., [31,32]), in which multiple words and objects are presented ambiguously in each trial and word-object pairings are derived by tracking co-occurrences across trials (e.g., [32]) and/or via top-down hypothesis testing mechanisms [33,34]. We note that our use of the term “explicit” in “explicit word learning” throughout the paper refers to the fact that in each trial in this task, word-object pairings are presented explicitly, that is, unambiguously, as each trial comprises a single word and a single referent. This is in contrast with XSWL, where word-object pairings in each trial are presented ambiguously, as two words and referents are presented in each trial, without indication of which word belongs to which referent. The term is not intended to specify the learning process by which the words are learned (i.e., via explicit vs. implicit learning).

With the goal of making only minimal changes to the laboratory-based tasks, we added a familiar-word practice test between the learning and test phases. Participants largely considered the experiment to be a strange novel game, experienced quickly in a local hut on the way to their main day’s work. In contrast, Experiment 2 took place as part of a major psychological and psycholinguistic “experiment fair” of four simultaneous experiments, for which the entire 30-household community took two weeks off from regular work. For this experiment, we focused our attention on the explicit word learning paradigm based on research showing better word learning performance in a similar explicit word learning task compared to XSWL [35]. Informed by the results of Experiment 1, we made several changes to the task to improve its suitability for testing with this population. These changes included informing participants of the purpose of the task from the outset, framing the experiment in a more real-world context, using colorful images of three-dimensional objects as our referents, familiarizing participants to referents ahead of time, and including non-verbal walk-through practice trials before the practice test.

## Experiment 1: Explicit vs. cross-situational word learning

In our first foray, we compared participants’ ability to learn novel word-object pairings in either an explicit word learning task, or a cross-situational word learning task. In both tasks there is a learning phase followed by a test phase. In the learning phase of an explicit word learning task, word-object pairs are presented individually, with one object visually presented simultaneously with its auditory label. While many new words are learned unambiguously like this, it is not the only way we learn words. When hearing an unknown word outside of a teaching context, it is not always clear what the referent of that word is. Cross-situational word learning paradigms test our ability to learn words in ambiguous scenarios (e.g., [31,32,36]). These paradigms begin with an ambiguous learning phase, in which more than one referent and/or auditory word is presented in each trial. In this way, it is not clear in an individual trial which word is associated with which referent, but over multiple trials, participants can derive the correct word-referent pairings by automatically tracking co-occurrence probabilities between words and candidate referents and forming associations between words and referents that co-occur with the greatest probability (e.g., [32]) and by hypothesis-checking techniques whereby the learner tests a possible word-object association by seeing whether the word and object co-occur in subsequent exposures (e.g., [33,34]). In both explicit and cross-situational paradigms, novel word learning is assessed in the test phase by showing participants more than one visual referent in tandem with one auditory label and asking participants to select the referent corresponding to the word.

Research into XSWL supports it as a viable mechanism for learning words in the real world. Adults (e.g., [32]), young children (e.g., [37]), and non-native learners [36] can learn words via XSWL in the lab, even when words only differ by a single consonant or vowel [31,38], and adults can retain these mappings over time [39]. In a direct comparison between explicit word learning and XSWL, participants who learned words via explicit presentation were more accurate at test compared to those who learned them via XSWL, though word learning for all groups was above chance [35].

Our aim was to use experimental tasks appropriate for use both in the laboratory and in remote communities, such that data collected would be valid in both contexts and comparable between them. Our approach was to find existing laboratory tasks that could be made suitable for fieldwork with minimal changes, to preserve comparability between both lab and field data. The simplicity of the tasks described above made them promising candidates for this purpose. Further, since there is no reason to believe that exposure to both unambiguous and ambiguous word learning scenarios is not a universal experience, and because the ability to form new word-object associations in these situations is thought to be via universal mechanisms of language learning, we believed these two tasks would be suitable across broader cultural contexts, despite being tested predominantly in WEIRD populations. That is, we hypothesized that learning in both scenarios should be universally possible, thus presenting a suitable platform to explore the ways in which our tasks may be unintentionally biased to a WEIRD audience in the case that they failed to demonstrate learning. As mentioned, while the word learning tasks described here are reasonably straightforward in their design, they make certain assumptions of participants that can safely be assumed in a Western, industrialized population, but not necessarily with groups of people unfamiliar with computer technology, digital media, and with the underlying assumptions of experimental psychology testing, which may be possible sources of bias should word learning not occur. For one, the tasks used here assume that the participant will recognize that the two-dimensional novel images presented on a screen represent referents that have (or have the possibility of having) associated auditory labels. Participants’ inexperience with computers may also interfere with their ability to learn information presented through this medium, and/or interfere with accurate registration of their responses, particularly with regard to more sensitive measures such as reaction time.

In this first experiment, we tested an isolated community in Papua New Guinea, basing our tasks and stimuli on those used in previous studies of explicit word learning (e.g., [40]) and XSWL (e.g., [36]), using auditory stimuli from [36] and visual stimuli from [40,41]. Unique to this version of the paradigm, we added a practice test between the learning and test phase using familiar words in the participants’ native language in an attempt to ensure participants were familiarized to the task.

### Method

#### Participants

Participants were 34 members of Towet village in the Saruwaged Mountains of Morobe Province, Papua New Guinea. Towet village is accessible only by foot (three days’ hike over the mountains to an urban center) or by small airplane. Photos of the village are available at osf.io/z4jt5. There is no electricity in the area. Cell phone coverage to the area began in 2015, but the signal was not strong enough to access the internet by cell phone. The nearest elementary school was established in 1998, and serves surrounding villages. Towet children must cross a log bridge over the roaring Uruwa River and hike up the other side of a steep river valley to reach the school. Education is mostly in Nungon for the first two years, then transitions to Tok Pisin, an English-based creole and a national lingua franca. People in the region are expert farmers and grow all the food they need, all year round. They eschew a market economy at the local level, preferring to maintain age-old traditions of sharing crop surpluses. In this regard, the region is exceptional, even within Papua New Guinea. They do, however, grow coffee for export; proceeds are primarily used to pay for school-related fees, including tuition and board outside the region for children who study beyond the eighth grade.

All participants spoke the Papuan language Nungon [42] as their first language. It should be noted that Nungon itself is an umbrella term for the speech varieties spoken in a dialect continuum in which each of six nearby villages has its own dialect. Most participants were native speakers of the Towet village dialect, but two were native speakers of other Nungon dialects who had married into the Towet community many years ago. Nungon speakers also traditionally have maintained trading relationships with distant communities who speak other languages. Due to this exposure to other Nungon dialects and other languages, most Towet Nungon speakers have some familiarity with or basic functional knowledge of, minimally, other Nungon dialects, and usually also some other languages or creoles, and thus experience with language learning. For instance, participants’ spoken knowledge of Tok Pisin varied from a few words to good communicative competence. Years in formal schooling ranged from none to eight; those participants who had gone beyond the fourth grade in formal schooling were also familiar with at least a few words of English.

While Nungon has a practical orthography, related to that of Tok Pisin, few participants were accustomed to writing in Nungon, and there were no books in Nungon at the time. Due to variable literacy levels, participant recruitment occurred via word of mouth, information and consent forms were translated and read aloud to participants, and instructions were given orally. Prior to participation, participants provided informed consent in accordance with the Australian National University Ethics approval, where the second author was located at the time. Fourteen participants completed the explicit word learning task (7 females, 7 males), and 20 completed the XSWL task (11 females, 9 males). While participants under 35 knew their birth year from written records, older participants gave an approximation of their birth year. Participants’ age ranged from 18 to 54 years in the explicit word learning condition (*M* = 38 years, *SD* = 13), and from 22 to 53 years in the XSWL condition (*M* = 33 years, *SD* = 10). For their participation, participants received 20 Papua New Guinean kina each: then approximately 6.07 USD. This was consistent with the second author’s usual pay rate for up to one hour of assistance with linguistic research. Since people do not pay each other locally for labor, there is no truly local equivalent rate, but we note that the Papua New Guinean official minimum wage is 3.20 kina per hour.

#### Materials

*Practice test stimuli*. Four Nungon words representing familiar items to members of the Nungon community (hut, grass skirt, tree, and airplane) were selected as practice test stimuli in order to teach participants how to complete test trials as well as confirm that they did understand the task instructions. These words were excised from recordings of native speakers of Nungon belonging to a Nungon adult speech corpus recorded in the course of general language documentation and grammatical description [42]. A photograph was selected to represent each word’s referent from the speech corpus’s accompanying photographic metadata.

*Test stimuli*. The eight novel auditory words (/fife/, /kɔko/, /kuke/, /pipe/, /popo/, /sase/, /sɛso/, /teko/) were selected from a set of words originally recorded for [43] and used in previous explicit word-learning [44] and XSWL [36] studies. The words were produced by a female native speaker of Brazilian Portuguese recorded at the Escola Superior de Propaganda e Marketing in São Paolo and adhered to Brazilian Portuguese phonology and phonotactics. Each auditory word was paired with a black-and-white line drawing of a nonsense object from [41], which were also used in [44] and [36]. The same word-object pairings were used for all participants.

**Setup and procedure.** Data were collected by the second author over a period of one week in April, 2017, during which time the researcher, who is adopted into the community, pursued additional research projects as well. Three local people, Stanly Girip, James Jio, and Lyn Ögate, who comprise the core of the researcher’s ongoing field research team on child language acquisition (e.g., [45–47]), served as assistants in running the experiment. This was their first acquaintance with the notion of psycholinguistic experimentation. Eventually, the three of them ran separate participants on the experiment simultaneously on the two laptops while the researcher looked on.

**Fig 2:**
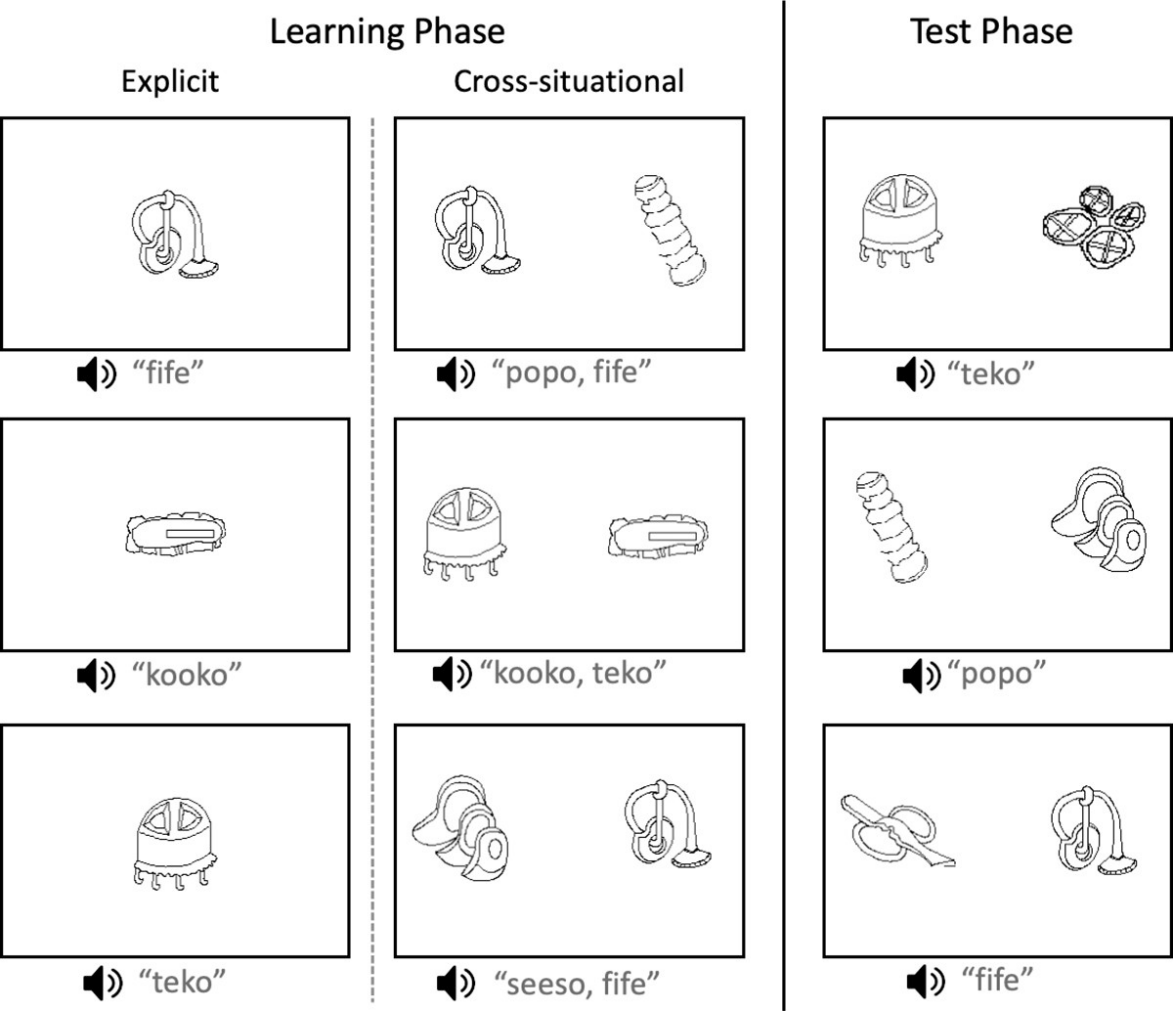
Examples of learning phase and test phase trials for Experiment 1. Participants completed either the explicit word learning or XSWL training condition.

Girip, Jio and Ögate recruited participants from among adult villagers. Participants were asked to participate in the study early in the morning, before heading out to their farms for the day. The study was run in a single hut with woven bamboo walls. Two Lenovo Ideapad 11.0-in laptops running PsychoPy version 1.85.1 [48] were placed side-by-side in the hut, mounted on a tarp that had been draped over rolled pandanus leaf mats. An image of the testing setup for Experiment 1 is available at osf.io/z4jt5/. Participants waited outside until they were called into the hut. Two people participated at a time when available. Participants listened to auditory stimuli through headphones belonging to the research team and generally used for linguistic transcription. All participants completed a learning phase, followed by a practice test and then the test phase. The entire session lasted approximately 20 minutes. Examples of learning phase and test phase trials are in Fig 2. Due to wide variability in literacy skills in the community, all instructions were given orally by Girip, Jio and Ögate.

*Learning phase*. The assistants running the experiment explained the initial task in Nungon, translating the following: “You will hear sounds and see images. Just watch and listen quietly.” Fourteen participants were run in the unambiguous, explicit training condition, and 20 participants were run in the ambiguous cross-situational training condition. Each trial of both learning conditions began with a 0.5 s ISI at which point the visual referent(s) would appear. In the explicit condition, the single visual referent was centered on the monitor. After 0.5 s, the auditory word corresponding to the visual referent would play with the trial ending 1.7 s after word onset. Participants were exposed to each word-object pairing seven times, resulting in 56 training trials, with the training session lasting approximately 2.5 mins.

In the XSWL condition, after the 0.5 s ISI, two visual referents appeared. These were centered vertically, and horizontally at 0.2 and 0.8 the proportional width of the display, for the left and right image respectively. This positioning was used every time an image appeared on the left and right throughout the experiment. After 0.5 s the word corresponding to one of the visual referents would play, with the word for the other referent playing 1 s after the onset of the first word, with the trial ending 2.5 s after onset of the second word. Each referent was paired with every other referent once such that participants were exposed to each word-object pairing seven times over 28 training trials played in random order. Whether a visual referent appeared on the left or right and which auditory word was presented first was counterbalanced across participants. In both conditions the learning phase lasted approximately 2 mins.

*Practice test*. Participants completed a familiar-word practice test before moving on to the test phase in order to teach them how to give responses in the task as well as gauge that they understood these instructions. Following the learning phase, participants were told the following, in Nungon: “Now, what you were hearing were actual words in another language. You are now going to take a test on what you’ve seen and heard. First, you will do an example test in Nungon, then you will do the actual test on the other language.” They were then instructed how to respond to the test images and sounds. After a 0.5 s ISI, in each of the four trials, participants saw a picture of two separate familiar items, with one positioned on the left and the right. After these had been on the screen for 0.5 s, the word corresponding to one of the items played. Participants were instructed to press the <a> key on a keyboard positioned in front of them if the word that they heard referred to the image on the left, or the <l> key if the word they heard referred to the right image. Most participants kept their index fingers resting above the <a> and <l> keys for both the practice test and subsequent test phase. Participants could respond as soon as the auditory word began, and the trial ended once they made a selection.

During the practice test, if a participant signaled that they did not understand the task (e.g., by asking the researcher what to do), the assistants and researcher helped those participants by talking through the trials until the participant understood what to do. That is, when the sound played, the research assistant would interact with the participant, asking what word they had heard, and then telling them, “So now press the key corresponding to the correct image.” Research assistants were asked not to tell participants the “right answer,” but were allowed to guide them in this way. Participants saw each practice trial once before advancing to the test phase.

*Test phase*. After the practice test, participants were told that they were now going to be tested on what they had learned from the first experience. They were told that they should give responses in the same way that they had with the familiar Nungon words in the practice test, but for the words of the new language they had heard in the first part of the experiment. Test trials followed the same design as practice test trials. Across the 28 trials, participants were exposed to each visual referent seven times, paired once with every other referent. Each word served as target three or four times, and this was counterbalanced across participants.

### Results and discussion

Data were averaged for each participant and analyzed using R version 4.0.2 [49]. First, we examined participants’ performance for practice trials. The proportion of correct responses was 0.86 (*SD* = 0.20), where 0.5 would reflect chance performance. A one-tailed, one-sample *t*-test confirmed their accuracy was above chance (*t*[33] = 10.69, *p* < .001, lower 95% CI [0.80]), suggesting that they had understood the procedure for identifying referents of already-known words.

**Fig 3:**
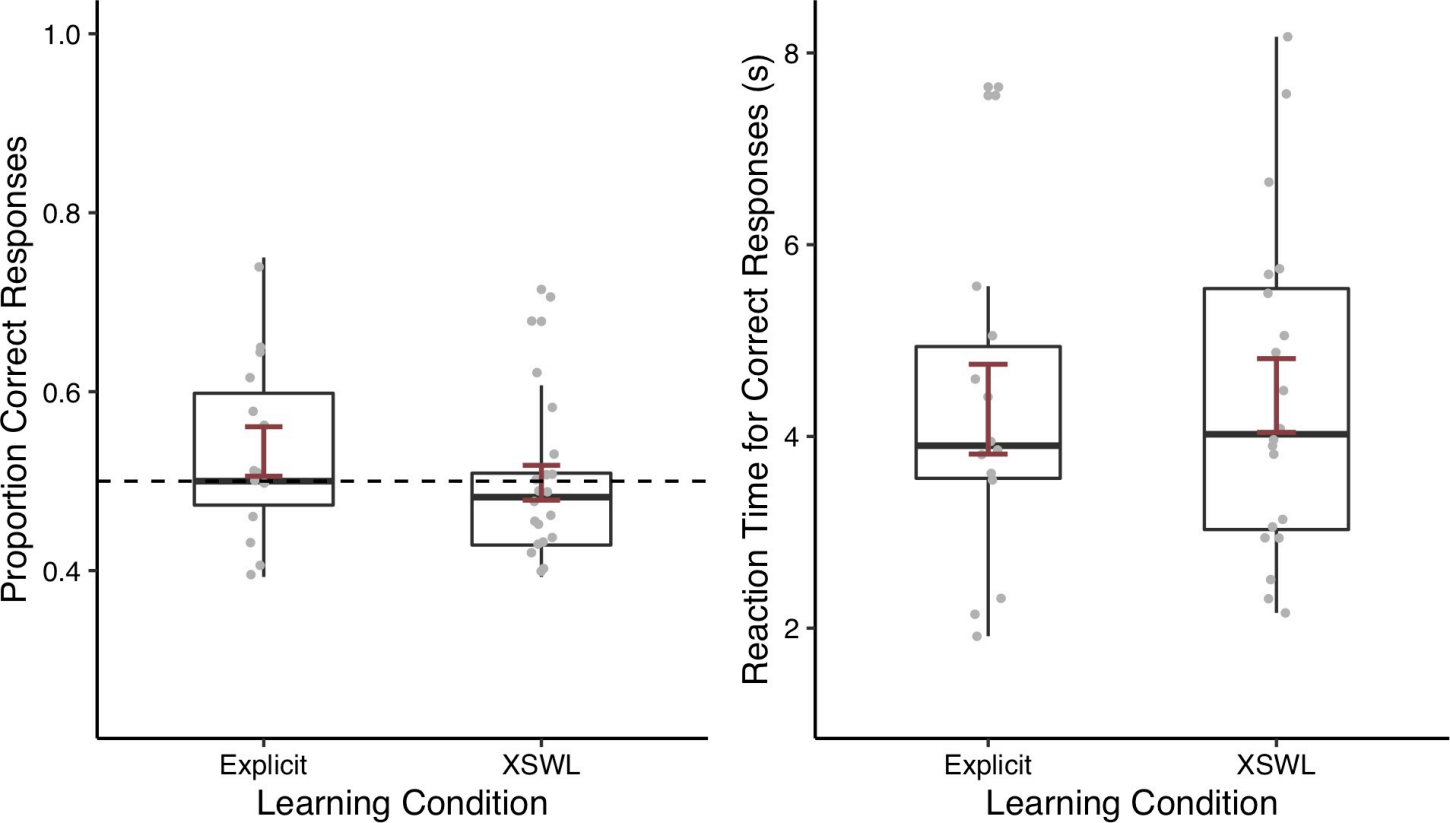
Proportion of correct responses (left) and reaction time (right) by participants who received training in the explicit learning condition or the ambiguous XSWL paradigm. Error bars represent one standard error.

To see whether participants were able to learn novel words via our tasks, and whether performance differed depending on whether word-object associations were presented unambiguously in an explicit word learning paradigm or ambiguously in a XSWL paradigm, we next looked at participants’ accuracy at test. We compared participants’ accuracy across word learning condition (explicit vs. XSWL) in an independent-samples *t*-test. As can be seen in Fig 3, there was no effect of learning condition (*t*[24.91] = 1.03, *p* = .311, 95% CI [-0.03, 0.10]), suggesting that performance did not differ depending on which learning phase participants experienced. One-tailed, one-sample *t*-tests against chance (0.5) for each learning condition were not significant, providing no evidence that participants learned the words in either the explicit condition (*M* = 0.53, *SD* = 0.10; *t*[13] = 1.20, *p* = 0.126, lower 95% CI [0.48]) or ambiguous XSWL condition (*M* = 0.50, *SD* = 0.09; *t*[19] = -0.09, *p* = 0.536, [0.46]). In contrast with similar lab-based studies with Western university students, in two studies of explicit word learning using the visual stimuli from the same set used here and a similar test phase in which participants chose between two possible referents, accuracy was reported as ranging from 71–100% [30], or at a mean of 93% [40]. Similarly, [36] report mean accuracies of 85% and 78% in two studies of XSWL that presented two words and items during learning and two possible referents at test, and which also used visual stimuli from the same set.

An independent-samples *t-*test on participants’ reaction time to correct responses also revealed no difference between learning conditions (*t*[27.79] = -0.24, *p* = .816, 95% CI [-1.38, 1.10]). As would be expected in a population with limited computer experience, participants’ reaction times appeared higher than values typically reported. On average, participants in the explicit learning condition took 4.28 s (*SD* = 1.75) to correctly respond to items, and similarly took 4.43 s (*SD* = 1.72) in the cross-situational condition. In contrast, [30], who used the same set of visual stimuli, reported an average reaction time of 1.28 s in their explicit word learning task, and [36, S1 Table] report average reaction times spanning 1.09–1.40 s across various conditions in their two XSWL experiments.

Thus, participants did not demonstrate word learning either when completing our explicit word learning task, or our XSWL task, and for both tasks showed longer reaction times than typically observed by Western participants. This could mean that they were unable to learn the associations between our novel words and line drawings, or alternatively that misunderstandings about the test phase of our experiment precluded the task from accurately capturing their learning.

**Fig 4:**
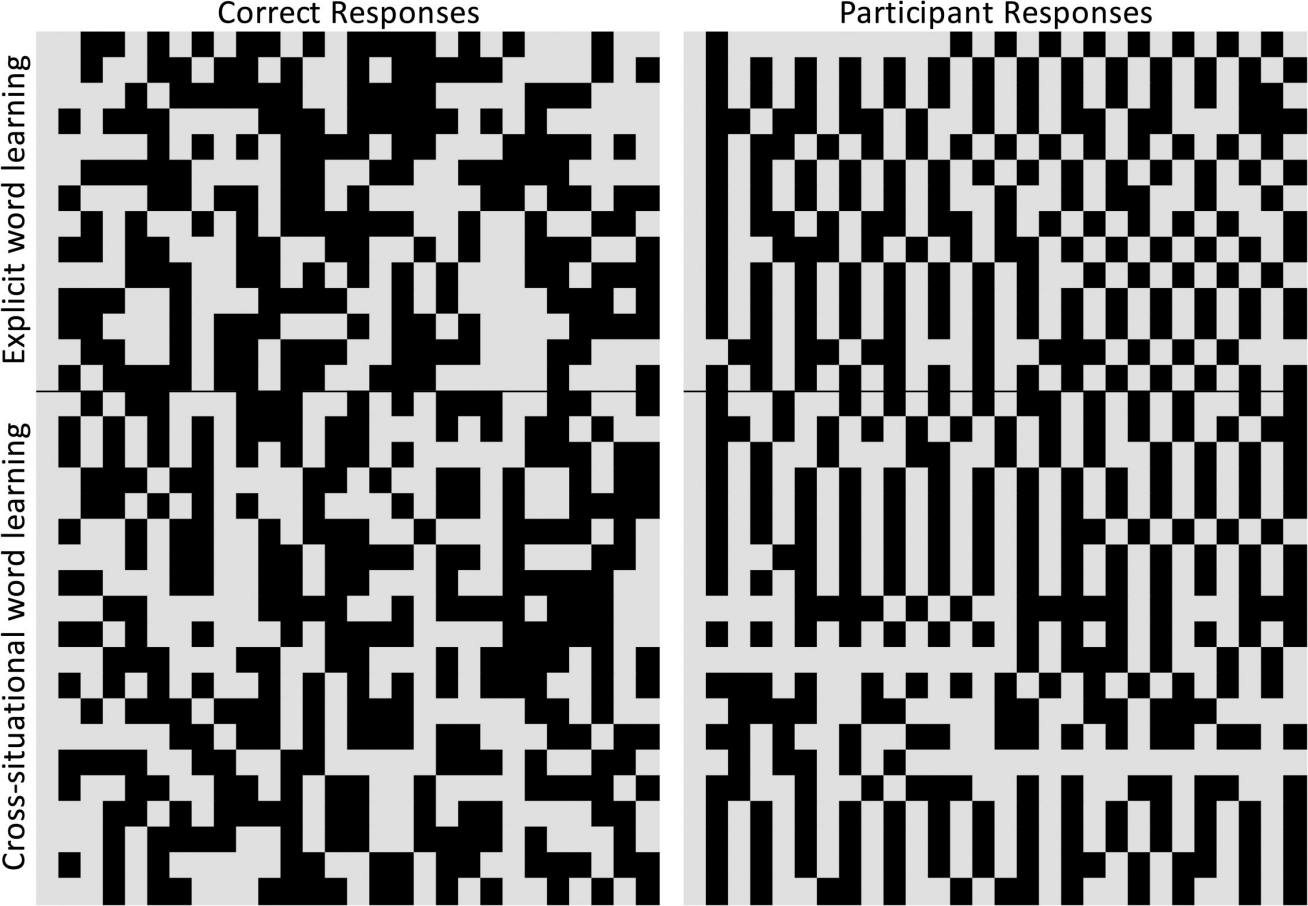
The left panel shows the randomized order of correct test responses. The right panel represents participants’ pattern of key responses, with each row representing an individual’s responses. Both panels are standardized such that the first correct key (left) or response key (right) is represented by gray squares, with the alternate key represented by black squares. While correct responses were randomized, many participants appeared to show a patterning to their responses.

Participants did appear to understand the practice test, selecting the correct photo referent associated with a known Nungon word 86% of the time. This would seem to suggest that their failure to identify the novel word referents at test may reflect failure to learn the words. However, as noted above, some participants did receive assistance from the researchers on some practice test trials, which may have inflated the practice test accuracy. As well, many participants appeared to give patterned or stereotyped responses at test. In Fig 4, each row of the left panel shows the pattern of correct responses across trials for one participant, that is, whether the named referent was on the left or right (standardized such that the correct side designation in the first trial is represented by a grey box throughout for that participant). Trial order was randomized for each participant, and this is reflected in the random appearance of the left panel. Participants’ responses in the right panel appear much less random, with many participants simply alternating keys each trial, or selecting one key for a stretch of trials. While these response patterns could simply reflect failure to learn the word-object pairings, it may also or instead reflect confusion with the test portion of the task, regardless of their apparent success with the known-word practice test.

Using this experience, in Experiment 2 we made changes to our experiment that we hoped would allow better assessment of participants’ learning in the task. These alterations were focused on the instructions and practice test, so as to not compromise the comparability of the learning phase and test portion of the task with results from a Western, laboratory setting.

## Experiment 2: Explicit word learning, revised

Because performance in Experiment 1 was at chance and did not differ between the two tasks, for Experiment 2 we limited our experiment to only one task. We chose the explicit word learning task, since participants in [35] demonstrated higher accuracy in the explicit condition than in XSWL.

We made several changes to our procedure in an attempt to clarify the task instructions to our participants, who were generally unfamiliar with computer technology and experimental behavioral testing. In Experiment 1, participants in both the explicit and XSWL conditions were not told until the test phase that they were completing a word learning experiment. Instead, during the learning phase they were instructed to simply watch and listen to the stimuli. Because one of the mechanisms purported to support XSWL is automatic statistical tracking (e.g., [32]), this approach is taken in some studies to mitigate participants’ use of more top-down associative strategies in forming word-object pairings [31,35]. Because Experiment 2 did not test XSWL, we were able to inform participants from the beginning that they would be taught novel words. We did this in the form of a mimicked real-world scenario. At the start of our task, participants were shown a picture of a woman (the “teacher”), surrounded by the novel visual referents used in the task. Participants were told that the novel referents were toys owned by the woman, and that she would like to teach them the name for each toy. The novel visual referents were replaced with colorful representations of novel three-dimensional objects used in previous studies of word learning (e.g., [31,35]). This allowed us to refer to the referents as objects owned by the women, which may have been less clear if using black and white line drawings as in Experiment 1. We also reasoned that providing adults with this short period of exposure to the visual referents at the beginning of the task may support success in the task (or at the very least would not hinder it), given that familiarizing young children to visual referents supports novel word learning, purportedly by reducing cognitive demands of the task [50].

We also reduced the number of learning phase trials in Experiment 2. In Experiment 1, each word appeared seven times during the training phase so that each word could be paired with every other word in the XSWL condition. Because we did not test XSWL in Experiment 2, we reduced the number of training trials from 56 to 24, such that each word-object pair was presented three times. While this may seem counterintuitive given that learning did not occur in Experiment 1, we felt having a smaller number of learning trials could help maintain interest in the task and limit fatigue.

Additionally, because it was unclear to what extent participants understood the task in Experiment 1, in Experiment 2, we developed two visual walkthrough instruction slides that appeared before the practice test and test phase to complement oral instructions.

### Method

#### Participants

Participants were 34 members of Towet village in the Saruwaged Mountains, Papua New Guinea. Prior to participation, participants provided informed consent in accordance with joint ethics approval from Australian National University and Western Sydney University, following the same approach as Experiment 1. Participants’ approximate age ranged from 18 to 55 years (*M* = 28 years, *SD* = 10). The birth year for one participant was not available, though the participant was estimated by researchers to be approximately 40 years old, well within the 18- to 55-year age range. Of the 34 participants, 15 were female and 19 were male. Participants were recruited via word of mouth in their community. Some of these participants would have also participated in Experiment 1 two years earlier. However, the exact number is difficult to verify due to approximations in age and spellings of names. For their participation, participants received 50 Papua New Guinean kina each, approximately 14.41 USD. This higher rate than Experiment 1 was consistent with the new rate set for participation in all the experiments run during this field trip. Data from one additional participant was excluded prior to analysis due to advanced age (78 years) relative to the other participants.

#### Materials

*Teacher images*. For the image of our “teacher” who would teach participants the names of the visual referents, we selected two photographs [51,52] of women from Papua New Guinea from a collection by the Australian Department of Foreign Affairs and Trade, maintained under a CC BY 2.0 license. Under this license, it is permitted to “copy and redistribute the material in any medium or format,” as well as “remix, transform, and build upon the material for any purpose,” (creativecommons.org/licenses/by/2.0). The images were cropped from their original backgrounds and placed on a blue background.

*Practice test stimuli*. Practice test stimuli were the same as in Experiment 1.

*Test stimuli*. Experiment 2 used the same auditory test words as Experiment 1, but a different set of visual referents. The eight visual referents were colorful pictures of novel items used in previous studies of word learning (e.g., [31,35,39]). Again, each auditory word was paired with a novel image, and all participants were exposed to the same word-object pairings.

#### Setup and procedure

The experiment was run as part of a diverse group of four psychological and psycholinguistic experiments brought to the region by four researchers. The researchers’ visit to Towet village represented a major event for the community. The second author’s long-term Towet collaborators, Stanly Girip, James Jio and Lyn Ögate, who had helped run the previous experiment, organized all aspects of this four-experiment suite and the researchers’ 11-day stay in Towet from late June and early July, 2019, through months of advanced planning. The community decided that all village adults would take two weeks off from regular farmwork and other duties, to be fully available for this community event to both participate in the experiments and provide support services, such as security and cooking for their visitors, and to serve as local organizers and research assistants. This meant that the entire community worked to stockpile foodstuffs, firewood, and chickens in the months leading up to the experiment fair.

An important feature of the experiment fair was the mentoring of young Towet adults who had obtained advanced education, by local standards. Organizers Girip, Jio and Ögate recruited four local research assistants who had obtained tenth grade or twelfth grade diplomas outside the region, but had failed to find further work or study opportunities, and returned to farm in Towet. These research assistants were trained in skills such as basic computer literacy, data entry, EEG electrode application, and eye-tracking methods, and also learned to run the experiments themselves.

The experiment fair was opened and closed with community gatherings, which all members of the Towet village community, along with invited guests from neighboring villages, such as the police representative from Worin village, attended. The researchers and a few local notables sat in a row on the lawn next to the purpose-built research building, while the research assistants sat in another area, and the organizers in a third area; they all faced the community, who sat on the grass or on porches on the periphery of the lawn. Both the opening and closing gatherings featured speeches by all the outside researchers that thanked the community and also explained their research projects, and speeches by community and religious leaders.

All experiments, including the current study, were run in different rooms of, or adjacent to, a new two-story building that had been purpose-built to house research and community training activities. The word learning experiment was run just outside this house, in an area with a large rectangular handmade table and benches enclosed by woven pandanus leaf walls and sheltered by pieces of corrugated roofing iron. Organizers Girip, Jio and Ögate managed participant flow to each experiment extremely efficiently, such that the only significant pauses between participants were during the researchers’ mealtimes.

The experiment was run on an Acer Swift laptop running PsychoPy version 1.85.1 [48], using headphones. After being trained by the second and third authors, local research assistant Ben Waum ran the experiment independently. All participants completed the learning phase, followed by a practice test and then the test phase. The entire session lasted approximately 30 minutes. Photos of the purpose-built building and testing setup are available at osf.io/z4jt5.

**Fig 5:**
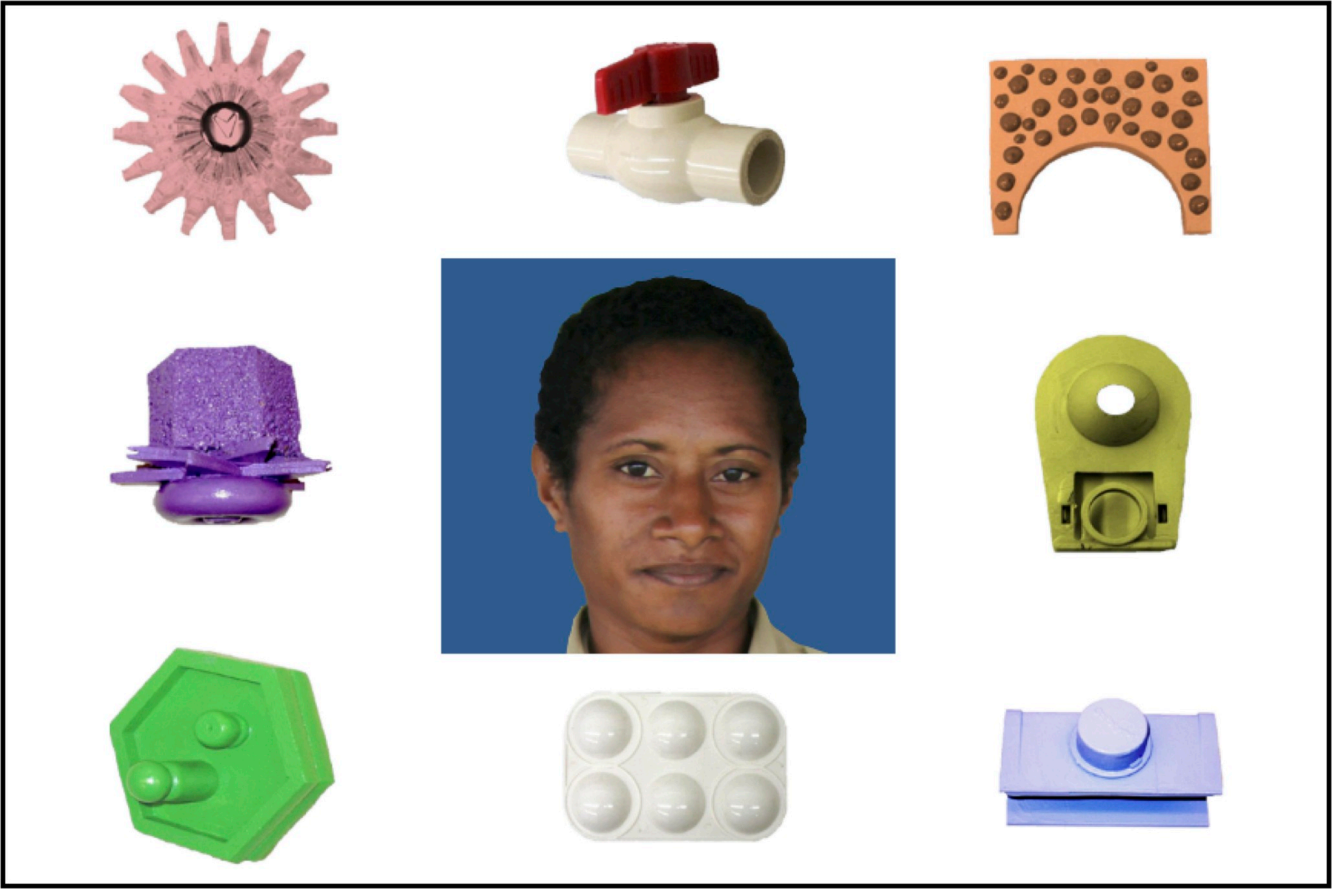
Example of the familiarization slide for Experiment 2.

*Learning phase*. Before the learning phase trials began, participants were first shown a picture of the teacher and were told by the researcher that the woman was from Papua New Guinea. After five seconds, the eight visual referents appeared around the teacher’s image (Fig 5), and participants were told that the woman had some small toys and would like to teach them what they are called. They were instructed that in this first part, they would see one image a time and the woman would say the name for that item, and that afterwards she would test them to see if they learned the words. The learning trials began once the space bar was pressed. The design of learning trials was identical to learning trials in the explicit condition of Experiment 1 in which one word and one image were presented in each trial, with the exception that the trial ended 2.5 s rather than 1.7 s after word onset. Participants were exposed to each word-object pairing three times, for a total of 24 learning trials. The learning phase lasted 1 min.

**Fig 6:**
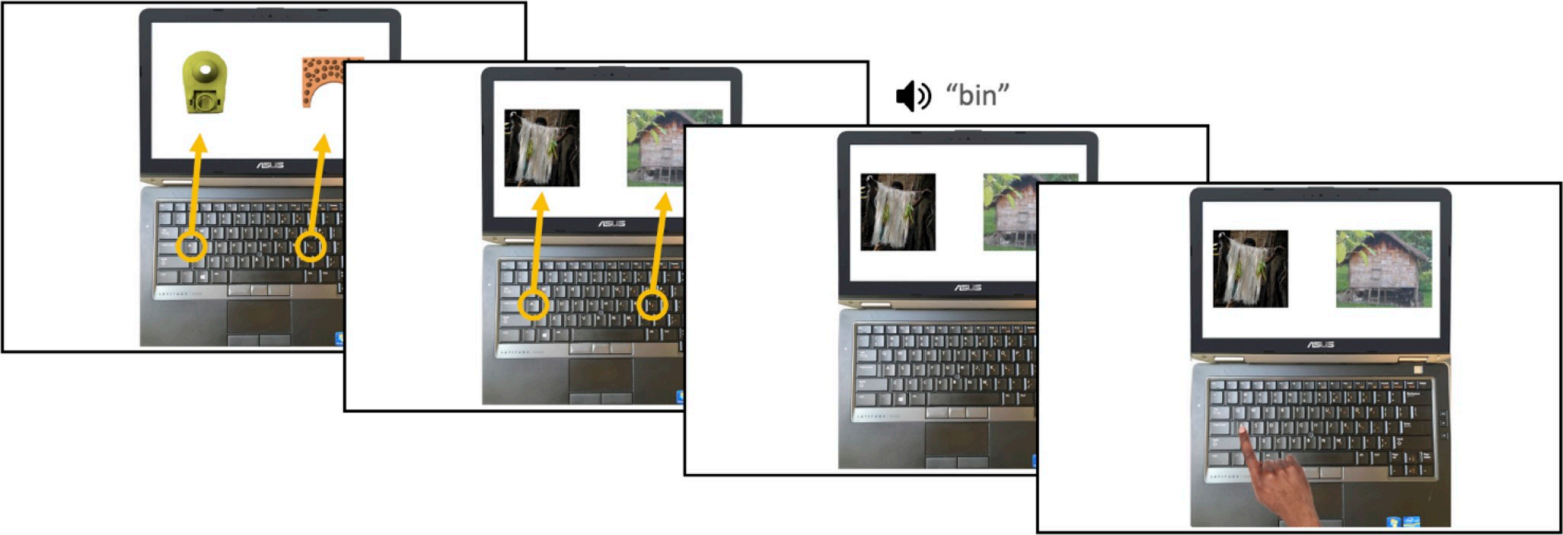
Example of the instructional slides preceding the practice test (left two slides) and a walkthrough trial (right two slides) showing participants how to pick the correct referent (grass skirt) for the Nungon word *bin*.

*Walkthrough trials and practice test*. Following the learning phase, the practice test was preceded by two example walkthrough trials (Fig 6), in which participants gave oral responses, but the experimenter entered their responses into the laptop. As can be seen in the first panel of Fig 6, before the familiar word walkthrough trials began, participants were first shown a picture of a laptop screen with two of the novel images appearing on the left and right, in order to illustrate what the test phase would look like. The <a> and <l> keys were circled with orange arrows pointing up to the image on the side of the screen they represented, to illustrate that each key corresponded to one of the images. While this picture was displayed, the experimenter explained to participants that for the test, they would be shown two of the women’s toys at a time, such as was currently on the screen in front of them. They were informed that the woman would say the name for one item, and that they should indicate whether they believed she named the item on the left or the right by pressing the key that corresponded to the left or right image. Participants were then instructed that they would first practice with some words they already knew. The two novel items were then replaced with familiar items (Fig 6, second panel). The orange arrows disappeared (third panel), beginning the first walkthrough trial, at which point participants were played the auditory word for the left item. An image then appeared of a finger pressing the <a> key on the laptop’s keyboard (fourth panel), corresponding to the left image. The trial advanced once participants indicated verbally that the word corresponded to the left image. The experimenter pressed the corresponding key, demonstrating how to respond, prompting a smiley face to appear. Participants completed a second walkthrough trial containing the same two referents, but hearing the word for the item on the right along with an image of a finger pointing to the <l> key. Once the participant gave the correct verbal response, and the experimenter pressed the correct key and cued the smiley face, they began the four practice trials. The practice trials were identical to those in Experiment 1. Because participants could potentially have learned to select <a> in response to the auditory word in the first walkthrough trial and <l> in response to the second by observing the keys the researcher selected in response to their verbal answers, when these words occurred as targets in the practice test, the location of the visual referent was switched relative to the walkthrough trials. As in the first experiment, the experimenter talked through practice trials with participants if they were unsure about what to do. While in Experiment 1 the experimenter would ask the participant which image corresponded to the familiar word, and then guide them towards selecting the key corresponding to that image, in this instance the community research assistant made the decision to enter responses for participants during the practice test until he was certain they understood the link between the keys and the pictures on the screen, at which point they entered their own responses. As in Experiment 1, participants saw each practice trial once before advancing to the test phase.

*Test phase*. The test phase began with presentation of the teacher’s image surrounded by the eight visual referents, and participants were told that the woman would now like to see whether they learned the names for her toys, and were reminded of the task instructions. To slow the pace of the test phase, the ISI was increased from 0.5 s to 1 s. Following this, two of the novel visual referents would appear on the left and right of the screen, centered vertically and centered horizontally at 0.2 and 0.8 proportional width of the screen, as in Experiment 1. After the images had been on the screen for 0.5 s, the auditory word associated with one of the images played. Participants pressed the key they believed corresponded to the spoken word. Once a selection was made, the next trial advanced. Participants completed 28 trials counterbalanced identically to Experiment 1 and presented in random order.

### Results and discussion

**Fig 7:**
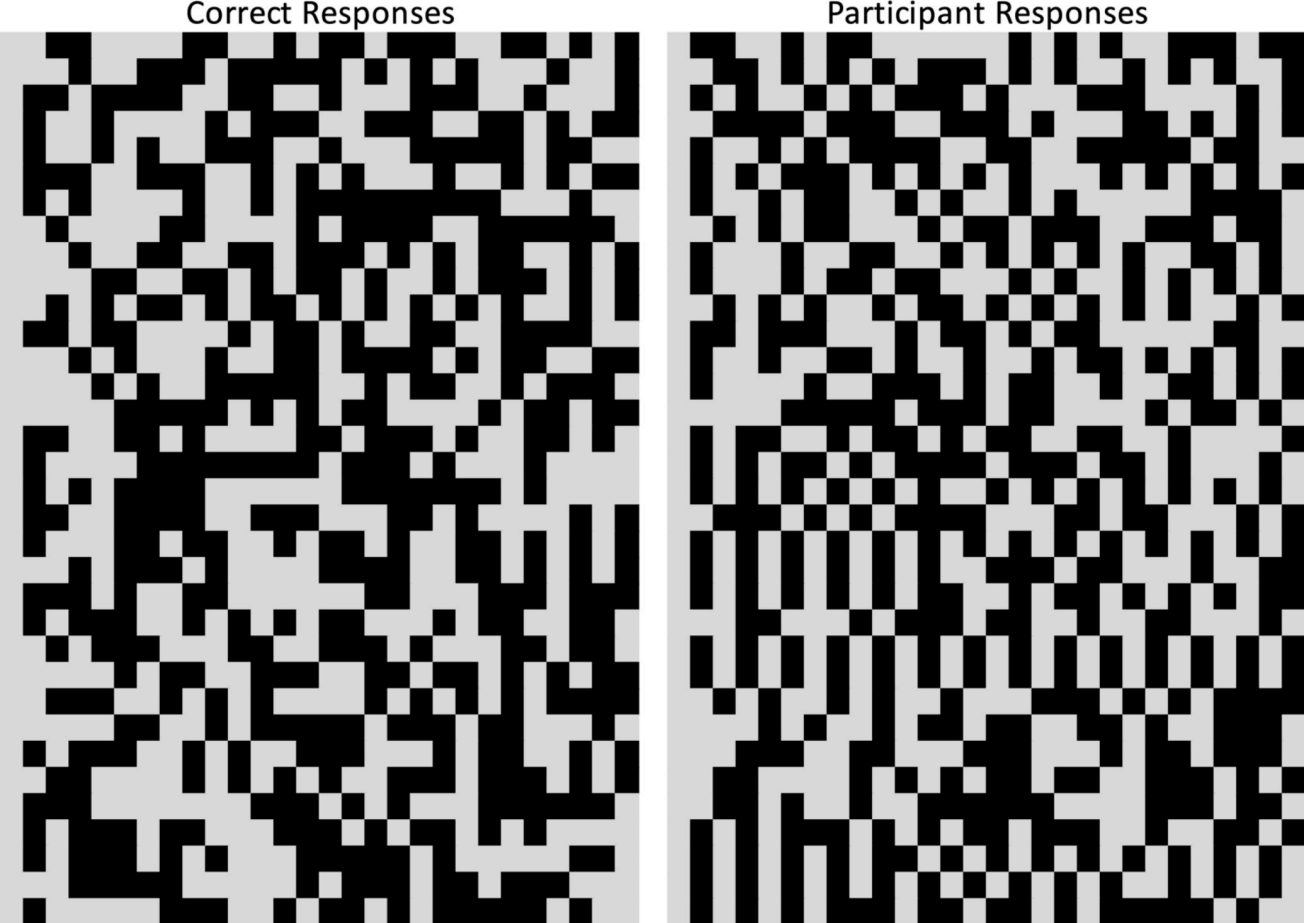
The left panel shows the randomized order of correct test responses. The right panel represents participants’ pattern of key responses, with each row representing an individual’s responses. Both panels are standardized such that the first correct key (left) or first response key (right) is represented by gray squares, with the alternate key represented by black squares.

As in Experiment 1, data were averaged for each participant and analyzed using R version 4.0.2 [49]. Visual inspection of participants’ responses at test appears to show fewer instances of participants giving patterned responses relative to Experiment 1 (Fig 7). Participants’ mean proportion of correct responses for practice trials was 0.94 (*SD* = 0.15) and was again above chance (*t*[33] = 16.98, p < .001, lower 95% CI [0.90]). However, as in Experiment 1, participants received assistance with the practice test as needed, which likely inflated accuracy.

**Fig 8:**
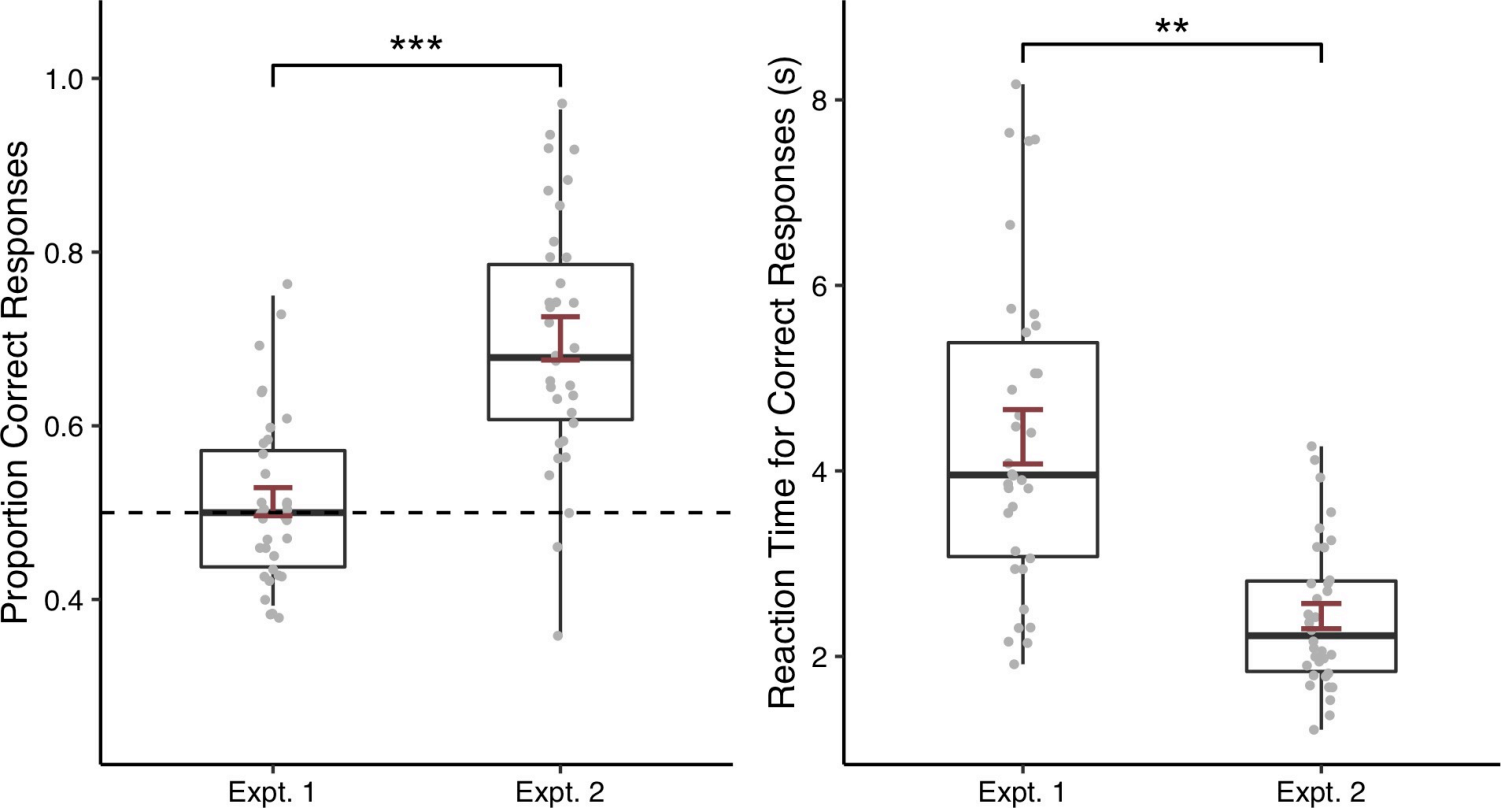
Proportion of correct responses (left) and reaction time (right) at test by participants in the explicit word learning condition in Experiment 1 and Experiment 2. Error bars represent one standard error. **p < .01; ***p < .001.

The proportion of correct responses at test was 0.70 (*SD* = 0.15). Unlike in Experiment 1, a one-sample, one-tailed *t-*test revealed accuracy at test to be well above chance (*t*[33] = 8.05, *p* < .001, lower 95% CI [0.66]). To see whether overall performance was improved in Experiment 2, we compared accuracy between the explicit learning condition in Experiment 1 with accuracy in Experiment 2 in an independent-samples *t*-test. As can be seen in Fig 8, participants in Experiment 2 did have a higher proportion of correct responses than participants in the explicit condition in Experiment 1 (*M* = 0.53, *SD* = 0.10; *t*[33.91] = 4.50, *p* < 0.001, 95% CI [0.09, 0.24]). This improvement in Experiment 2 was despite participants receiving a greater number of learning trials in Experiment 1. Likewise, reaction times for correct responses were faster in Experiment 2 (*M* = 2.43, *SD =* 0.79) compared to the explicit condition in Experiment 1 (*M* = 4.28, *SD* = 1.75; *t*[15.24] = 3.79, *p* = 0.002, [0.81, 2.89]; Fig 8). Notably, while mean accuracy here was still below the 93% reported by [40] in their laboratory-based explicit word learning task, it approached the lower end of the range of observed values reported by [30] (71%). And while the mean reaction time of 2.43 s here in Experiment 2 was still longer than the average 1.28 s observed by [30], it fell below the range of means observed in Western university students by [35, Table 3] in their explicit word learning condition (2.59–3.08 s), which used the same set of visual stimuli as Experiment 2. Though [35] offered students four options at test compared to two options here, the values are nonetheless comparable.

Thus, it appears that our manipulations in Experiment 2 led to successful word learning and were able to capture word learning at test in members of this remote, isolated community. Unlike in Experiment 1, participants’ overall word learning accuracy was above chance, and was significantly improved relative to the explicit learning condition in Experiment 1. As well, relative to Experiment 1, participants’ responses appeared to have fewer instances of patterned responses in Experiment 2.

### General discussion

With twin goals of encouraging research in a more diverse range of peoples and of empowering such communities to become partners in these endeavors, we set out to adapt a laboratory word learning task for testing in a remote, understudied population. We tested word learning in a relatively isolated community in Papua New Guinea, whose members had minimal familiarity with computer technology and behavioral experimental test-taking. Our aim was to adapt the task in a way that maintained the integrity of the learning and test phase, leaving the potential for future comparisons between participants from a diverse range of societies, both industrialized and not.

To that end, in Experiment 1 we designed a cross-situational word learning task and an unambiguous, explicit word learning task based on previously run experiments (e.g., [31,35]) and using stimuli from other studies on word learning [36,40,41], comparing word learning between the two tasks. We wanted to see if our participants, who had minimal-to-no prior experience with computers or experimental testing, could nonetheless successfully perform in our task with minimal modifications. Thus, the only adaptation we made relative to our laboratory-based task was to add a practice test phase after the learning phase but before the test phase. This comprised four trials following the same format as the novel word test trials, using familiar words from the participants’ native language, and using photographs as visual referents. Despite accuracy for the practice trials being well above chance, participants failed to demonstrate word learning in either the explicit or cross-situational task, with performance not different from chance. We observed a patterning of responses by many participants, suggesting that word learning did not occur and/or that participants may not have understood the task.

Since performance did not differ between tasks in Experiment 1, in Experiment 2 we chose to focus our efforts on only one word learning task. We chose the explicit word learning task, as [35] found better word learning in their explicit word learning condition than their XSWL conditions, using similar designs. We made several additional changes to our experimental task to try and elicit learning from our participants. Some of these changes were centered around helping participants understand what the testing phase was asking of them, and how to respond to trials. As in Experiment 1, we included familiar word practice test trials prior to beginning the testing phase. In Experiment 2, these were additionally supported by nonverbal supplementary instruction slides that included two walkthrough practice test trials. Additionally, due to convention in the XSWL task, in Experiment 1 participants were not informed that they were completing a word learning task until after the learning phase. In Experiment 2, participants were told from the beginning that this was a word learning task, described in the form of a “real-world” scenario, in which a woman had several new objects and wanted to teach the participant the word for each object. To that end, we also replaced the two-dimensional line drawings from Experiment 1 with colorful images representing three-dimensional objects.

While we recognize that the entirety of the experience was not a part of natural, everyday life for our participants, some of our changes attempted to improve the naturalness of our task. Relating the aim of our task in the form of a “real-world” scenario with a “teacher” was one such example; as mentioned, Towet community members do commonly experience learning to communicate with people who speak other Nungon dialects or other languages. Replacing black-and-white line-drawn visual referents with colorful drawings representing three-dimensional physical objects also may have more closely resembled a real-world experience. We note that using photos of real objects whose labels were unknown to participants could have further enhanced the real-world context of our study. However, our goal here was to still use stimuli that has been used in previous word learning studies [31,35].

Additional changes were made in the hope that they might ease general cognitive demands in the task and help support or unmask any learning. Participants were briefly familiarized to the images of the visual referents prior to the learning phase and again prior to the testing phase. Familiarizing participants with the novel visual referents prior to completing a word learning task has been shown to support word learning in young children [50]. While [50] used a prolonged exposure period spanning weeks of in-home exposure, we reasoned that even a quick familiarization to the visual referents prior to completing the task may ease subsequent visual processing demands when seeing the images again. We additionally reduced the number of training trials from 56 trials in Experiment 1 to 24 trials in Experiment 2, reducing the number of times participants were exposed to each of the eight words from seven times to three times. While one might think to increase the number of training trials when learning has not occurred, the number of exposures to each word was initially at seven in order to match the number in the XSWL condition, and we were concerned that having too many learning trials may tax participants’ sustained attention.

Unlike in Experiment 1, in Experiment 2 participants did demonstrate word learning, suggesting our manipulations may have been successful either in supporting word learning or enabling participants to demonstrate their learning. Participants’ overall accuracy was above chance, and was greater than accuracy in the explicit word learning condition in Experiment 1. As well, there appeared to be fewer instances of patterned responses in Experiment 2 compared to Experiment 1. These findings might suggest that efforts aimed at improving participants’ understanding of the task may have been successful in that regard, but in the absence of systematic testing of these factors, this is only speculative.

We emphasize that at this point it is not clear specifically which manipulations were beneficial, or more beneficial than others. The time and resources required to arrange and conduct testing in a remote location such as this rendered a more systematic approach impossible, and after the failure to find evidence of word learning in Experiment 1, we chose to maximize our efforts in Experiment 2. However, as more efforts are made to adapt laboratory-based tasks for testing in broader cultural settings, a clearer picture of which types of manipulations are most helpful will likely emerge. For instance, [53,54] explored the perception of gender marking in Konso by native speakers located in a village in Ethiopia using a computer-based task. As in the current experiment, participants had limited experience with computer technology, and no prior experience with experimental psycholinguistic testing. Similar to findings here, in their initial experiment the authors suspected that this inexperience masked participants’ ability to perform in the task, as reaction times were markedly slower than what is typically observed, and some participants appeared unsure of the task instructions during the experiment. This led the experimenters to alter their practice test so that it very closely mirrored the experimental test, and to give oral instructions only in Konso, rather than at times using Amharic, a lingua franca of the region. These manipulations were successful in reducing participants’ reaction times and improving their understanding of the task such that the researchers were able to uncover meaningful patterns of performance.

In another instance, [55] describe an attempt to run a statistical word segmentation task (based on [56]) in another community in Papua New Guinea. In this type of task, participants listen to strings of nonsense speech that contain “statistical words” that can be derived by automatically tracking the probability that a given syllable follows another. In this way, higher transitional probabilities cue syllables belonging to the same word, and lower probabilities between syllables cue word boundaries [57]. With a similar goal of helping to ensure participants understood the task, the experiment was preceded by a practice experiment in which participants were familiarized to strings of rising tones and were then played a rising and falling tone. They were asked to select which tone was “good” (this wording was used as an approximate translation given the vocabulary of the language) based on what they remembered hearing in the string of tones. Participants also received feedback and instruction from the experimenter and research assistant. Despite this, the experimenters were unable to uncover evidence of statistical word segmentation in their experiment.

While there were many differences between these tasks and ours, this appears to underline the importance of including a meaningful practice test that can be clearly understood by participants when adapting tasks for populations less familiar with computer technology and/or experimental testing. Comparing the manipulations between [55] and our study may more specifically point to presenting the practice test immediately before the experimental test, and/or using familiar words in the practice test, as manipulations to be given further consideration. As well, consultation and collaboration with community members in identifying problems or points of confusion in existing tasks and coming up with possible solutions would likely streamline the process of adapting tasks to broader communities.

Of course, we note that the adaptations we made to our explicit word learning task would not be specifically appropriate for a wide array of tasks. For instance, even though our testing phases were identical between our explicit word learning and XSWL tasks, not all of the manipulations made to the explicit word learning task in Experiment 2 would have been appropriate to apply to the XSWL task. For one, while XSWL is purported to be supported both by automatic statistical tracking (e.g., [32]) and top-down hypothesis-checking mechanisms (e.g., [33,34]), it is conventional for researchers wanting to reduce the influence of top-down mechanisms to not inform participants of the task objective until after the learning phase. Thus, this would limit the ability to present the task in our context of a real-world scenario in which the woman wanted to teach the participant the names of her novel items. Even if limiting top-down influence was not a concern for the researcher, participants may have found it confusing as to why the woman would go on to present the words in an ambiguous way. Instead, we hope that researchers employing a range of tasks that measure a range of human behaviour and cognition can take away the themes of our adaptations and apply them to their own tasks as appropriate.

Relatedly, in addition to certain laboratory tasks perhaps being more amenable to adaptation than others, certain paradigms may be more appropriate for testing across broader cultural contexts than others. As mentioned in the introduction, there is considerable cultural influence in the way in which many laboratory tasks have been designed. In addition to assuming comfort with technology in many instances, they also assume familiarity with a test-tasking style of task, and may ask questions that may be seen as culturally inappropriate in non-Western settings, for instance in asking about opinions on something not directly experienced by the participant [29]. As pointed out by [55], many laboratory-based paradigms rely on participants making metacognitive, or in the case of psycholinguistics, metalinguistic judgments. The authors of [55] speculate that the ability to make such judgments may be culturally influenced rather than a human universal, and may in part underlie their inability to capture statistical word segmentation in a non-WEIRD population in their task. Indeed, the growing exposure to and experience with these types of paradigms and the questions they ask may have also contributed to our successful capture of word learning in Experiment 2. Experiment 2 represented many participants’ second, third, or fourth time participating in a psychological experiment run from a laptop, since Experiment 2 was part of a suite of experiments offered to the Towet village community, and some participants in Experiment 2 had likely also participated in Experiment 1, two years prior. The Towet organizers of the experiment fair had run Experiment 1 without any familiarity with the goals and expectations of these sorts of “tests” two years earlier. During the months in which they worked with the community to plan for the researchers’ visit, they and the community would have had time to reflect on their experience in Experiment 1, and mentally prepare for more of the same in Experiment 2 and the accompanying new experiments.

While this type of repeated and relatively extensive exposure to experimental behavioral testing may have enhanced participants’ familiarity with technology, laboratory paradigms, and perhaps their ability to make these types of metacognitive judgments, fieldwork—particularly in remote communities—is extremely time and resources intensive, and thus would likely not be possible in many situations. One possible solution is to instead use methods that reduce the indirectness of responses. For instance, asking participants in our task to select the correct referent on a touchscreen, or asking them to select actual objects presented in front of them would have removed the barrier of having to associate peripheral keyboard presses with images presented on a monitor. As well, future approaches could request less overt responses from participants, for instance by asking participants to name an object or repeat a phrase (as suggested by [55] and implemented in [58]), rather than make a decision about which item may be correct or incorrect in a forced-choice task. And methods that measure more automatic, subconscious behaviours, such as eye-tracking and EEG, would be expected to be less affected by cultural influences. Indeed, one of the four experiments included in the fair was an eye-tracking study measuring eye-voice span [59] to inform when Nungon speakers plan their sentences. In this case data was elicited through the natural task of asking participants to describe images and tell stories in their own language. A further two experiments implemented EEG, with data collected through having participants passively listen to speech while watching a silent nature documentary (as in [60,61], in which participants were tested in a Western laboratory setting). These studies are currently under review or in preparation for publication.

Additional lessons from our endeavor concern the implementation of the testing and recruitment. Participants in Experiment 1 lined up before a small hut (belonging to a local person) in the early mornings to participate quickly, before heading off to their “real” work for the day; they earned 20 kina each. In contrast, participants in Experiment 2 took off two weeks from their regular duties to help with the experiment suite to which Experiment 2 belonged, which was run in a large, purpose-built building known locally as “the office.” Not only did they earn 50 kina per experiment in which they participated, but many earned additional money through employment as cooks, security, and porters for the researchers and local organizing team; organizers and research assistants were also paid. Unlike with Experiment 1, for which research assistants had some difficulty recruiting busy participants, participants for Experiment 2 were available and ready to participate throughout each day, such that as soon as one participant finished the task, another would be summoned. The experiment fair also captured the attention of local people who previously had not shown much interest in the second author’s ongoing linguistic research in the area. Community leader Mark Girip told Author 2 in the closing ceremony: “Before, when you used to come here alone, I thought your work was inconsequential. But this time, when you arrived, followed by a long line of others, I realized that you are doing major work.”

The construction of a purpose-built facility and the considerable planning efforts the community undertook to prepare for the experiment suite that included Experiment 2 must be attributed in part to the long-standing research collaboration between the second author and the Towet village community, which has resulted, among other research products (e.g., [46,47,62]), in Nungon becoming the first indigenous Pacific language to enter the CHILDES database [45]. Such a massive community effort might be difficult to summon without a preestablished personal connection with the community, hence we advise that those wishing to do psycholinguistic experimentation in a field setting collaborate with community members or others who have long-term connections with communities.

Long-term relationships may be an essential component of fair benefit-sharing, and maximizing of the participatory character of this sort of research. Short-term research projects are disappointing for communities that yearn for continuing connections [63], not unlike Towet villagers’ generations-old trade-friend relationships with distant villages along their traditional trade routes. The second author’s adopted brother in Towet once called her a “bridge” to the outside world for his community; researchers who drop into a community to run one experiment, then disappear forever, cannot serve as bridges. Although the experiments described here were planned and designed overseas, local research assistants were encouraged to engage with the presenting technology and were trained in computer skills. In the closing ceremony after the conclusion of Experiment 2, Ben Waum, the research assistant who single-handedly ran Experiment 2, said (in Nungon), “I’ve lived outside this area for a long time, gone to school in the city, and done various types of work. So it is amazing that the first time that I touch a computer happens to be when I am back in the village. Having tasted this sort of work, my insides are burning with the desire to do more.”

It is our hope that our experience and detail of the adaptations made to our laboratory-based experimental word learning task can inform application of similar adaptations to other commonly used laboratory tasks to render them more appropriate for testing in a wider variety of people. To that end, our task and data are available (osf.io/z4jt5) for researchers to use. Having established a word learning task suitable for testing in the field, ongoing work in our lab explores the effect of specific versus general outgroup biases on word learning from “teachers” of various ethnic backgrounds, as members of remote communities would not be expected to form specific biases to the same extent given their minimal exposure to people outside of their community. As well, switch-reference marking [64] is a unique morphosyntactic feature of the Nungon language (along with many Papuan languages) in which it is indicated in advance whether the subject of an upcoming clause will differ from that of the current clause. This may entail advanced multi-clause predictive and planning processes on the part of Nungon speakers [46]. While researchers have begun to explore some of the psycholinguistic effects of this feature during the “experiment fair” using eyetracking and EEG methods, the ability to adapt overt response tasks for the remote field expands the breadth of questions that can be asked about how this unique feature shapes language learning and processing, and how it may affect more general cognitive processes.

To conclude, the majority of psychological research, including psycholinguistic research, has been conducted on a limited set of the world’s population, primarily focusing on people from western, industrialized societies [1], and in the case of psycholinguistics, the languages they speak. This has limited the interpretability of existing research and restricts us from discovering and understanding the full range of human diversity. Only through efforts to expand testing to members of understudied societies can we gain a more complete picture and understanding of our human features and capabilities. Of course, the burden of expanding experimental testing to a broader cultural audience doesn’t lie solely on adapting Western-based tasks to a non-Western audience. Researchers may consider reducing the ingrained cultural aspects of tasks presented to a Western audience as well, which may more accurately capture core human behaviours and cognitions, and allow for better comparison across cultural contexts.
